# Comparative effects of epidural analgesia and intramuscular morphine on maternal and neonatal outcomes: a retrospective cohort study

**DOI:** 10.1016/j.xagr.2024.100324

**Published:** 2024-02-23

**Authors:** Abdelrahman Elsayed, Ismail Abdelhady, Fawzia M. Elgharbawy, Ashraf Gad

**Affiliations:** a College of Medicine, Qatar University (Dr Elsayed); b Division of Neonatology, Women's Wellness and Research Center, Department of Pediatrics, Hamad Medical Corporation (Dr Abdelhady and Dr Gad); c Division of Neonatology, Department of Pediatrics, AL Wakra Hospital, Hamad Medical Corporation (Dr Elgharbawy) Doha, Qatar; d Department of Pediatrics, Weill Cornell Medicine-Qatar (Dr Abdelhady, Dr Elgharbawy, and Dr Gad), Doha, Qatar

**Keywords:** epidural analgesia, morphine, neonatal intensive care unit, neonate, prolonged labor, respiratory distress

## Abstract

**BACKGROUND:**

The global practice of pain management during labor involves the use of epidural analgesia or intramuscular morphine. However, the impact of these methods on maternal and neonatal short-term outcomes remains uncertain.

**OBJECTIVE:**

This study aimed to evaluate the effect of labor exposure to epidural analgesia and intramuscular morphine on neonatal intensive care unit admission rates and other associated maternal and neonatal outcomes such as sepsis, respiratory distress, instrumental delivery, birth trauma, low Apgar score, and chorioamnionitis.

**STUDY DESIGN:**

A study at the Women's Wellness and Research Center in Qatar analyzed 7721 low-risk normal vaginal deliveries from January 2017 to April 2018. Results were analyzed using descriptive and backward stepwise multinomial regression analysis, categorizing outcomes on the basis of pain management during active labor.

**RESULTS:**

Of the 7607 participants in the final sample, 2606 received epidural analgesia, 1338 received intramuscular morphine, 286 received both, and 3304 received neither. Multinomial regression analysis revealed no difference in neonatal intensive care unit admission in the epidural analgesia group or in the intramuscular morphine group compared with the group that received neither intervention. However, the analysis showed a significant association between the combined use of epidural analgesia and intramuscular morphine and neonatal intensive care unit admission due to respiratory depression (adjusted odds ratio, 8.63; 95% confidence interval, 1.07–69.46; *P*=.04). Moreover, there was a significant association between prolonged duration of the second stage of labor and receiving epidural analgesia alone (adjusted odds ratio, 1.02; 95% confidence interval, 1.01–1.02; *P*<.001) or the combination of epidural analgesia and intramuscular morphine (adjusted odds ratio, 1.02; 95% confidence interval, 1.01–1.03; *P*<.001). In addition, the combined use of epidural analgesia and intramuscular morphine was associated with gestational age (adjusted odds ratio, 1.86; 95% confidence interval, 1.19–2.90; *P*=.01) and infant sex (adjusted odds ratio, 3.72; 95% confidence interval, 1.54–9.01; *P*=.003). Intramuscular morphine alone was only linked to low Apgar score at 1 minute (adjusted odds ratio, 6.29; 95% confidence interval, 1.33–29.83; *P*=.02).

**CONCLUSION:**

In low-risk mothers, combining epidural analgesia and intramuscular morphine during labor increases NICU admission risk due to respiratory depression. However, the individual use of either method shows distinct clinical profile. Further research is warranted to enhance understanding and optimize pain management protocols.

**AJOG Global Reports at a Glance**

**Why was this study conducted?**

This study was conducted to compare the effects of different pain management methods during labor on various maternal and neonatal variables in low-risk pregnancies.

**Key findings**

The use of epidural analgesia (EA) alone or intramuscular (IM) morphine alone is not associated with increased risk of neonatal intensive care unit (NICU) admission. However, EA use was associated with prolonged labor, with no association observed with increased rates of operative vaginal delivery. The combined use of the 2 methods was associated with increased risk of NICU admission due to respiratory depression and neonatal acid-base derangements.

**What does this add to what is known?**

There are limited data on the safety of the combined use of EA and IM morphine for pain control during labor. Our results indicate that their combined use requires caution until more evidence is available.

## Introduction

Neuraxial anesthesia (NA) is widely recommended for labor pain management, providing effective relief during the first and second stages of labor. It also helps control maternal pain during delivery, a peak of pregnancy-related stress. In addition, early neuraxial catheter installation is advised for high-risk patients to reduce the need for general anesthesia and its negative consequences.^2^ Despite the great benefits of NA, its effects on short-term neonatal complications are uncertain. Multiple studies have shown that epidural analgesia (EA) is associated with a prolonged second stage of labor and an increased risk of instrumental delivery,^3^^,^^4^ increased maternal fever,^5^ lower Apgar scores,^4^, ^5^, ^6^ and neonatal intensive care unit (NICU) admission.^5^, ^6^, ^7^ Conversely, one study found that neonatal resuscitation and NICU admission were not affected by EA use.^4^ Another study suggested that the mode of delivery mediates NICU admission and neonatal resuscitation needs, not EA use.^8^

Opioids offer a viable alternative for deferred EA because of their widespread availability, cost-effectiveness, and safety. However, adverse effects on maternal and neonatal health have been reported.^9^ One trial comparing intramuscular (IM) morphine with IM pethidine found that IM morphine had a longer duration of efficacy for pain control.^10^ Another trial comparing remifentanil to IM pethidine showed that women who received remifentanil had better pain tolerance during labor.^11^ A trial comparing fentanyl to pethidine concluded that fentanyl is a better choice for pain relief, with less sedation, shorter labor duration, and fewer difficulties in establishing breastfeeding.^12^ Although various opioids have been compared in different studies, to the best of our knowledge, none have investigated the effects of administering both EA and IM morphine during labor on neonatal or maternal outcomes compared with no intervention or EA alone.

### Study objective

This study aimed to compare the effects of using either EA alone, IM morphine alone, or both on NICU admission rates. The secondary objectives included neonatal and maternal short-term outcomes.

## Materials and Methods

### Study design

This study analyzed nulliparous and multigravid mothers with low-risk singleton pregnancies who underwent normal vaginal delivery between January 2017 and April 2018. The mothers were categorized into the following 4 groups based on pain management during active labor: Group 0, including patients who neither received EA nor opioids during labor; Group 1, including those who had EA intrapartum with no IM morphine; Group 2, including those who received IM morphine but did not receive EA; and Group 3, including those who had both EA and IM morphine.

### Pain-relieving regimens

Patients were administered pain management regimens based on local recommendations from the Women's Wellness and Research Center (WWRC), previously known as the Women's Hospital. Per the local recommendations, all patients in active labor are recommended to have EA as the main method of pain management during labor. For patients receiving EA, a low-dose fentanyl/levobupivacaine mixture (0.1% levobupivacaine with fentanyl 2 mcg/mL) was used to initiate analgesia. The initial loading dose ranged from 15 to 20 mL, followed by a programmed intermittent epidural bolus of 10 mL every 45 minutes in conjunction with patient-controlled epidural analgesia that had a bolus of 5 mL and a lockout interval of 10 minutes. In cases where neuraxial blockade was not suitable or declined by the mother, IM morphine was offered. Patients received an IM injection of up to 10-mg morphine, with the possibility of repeat injections every 4 hours if necessary.

### Inclusion and exclusion criteria

This study considered the medical records of women who underwent a normal vaginal delivery or assisted vaginal delivery at term, between 37 and 42 weeks of gestation, from January 2017 to April 2018. Eligible women were those who carried a singleton pregnancy with no known comorbidities or complications during the prenatal period. The exclusion criteria included multiple gestations, labor occurring in either the preterm (<37 weeks) or postterm period (>42 weeks), elective or emergency cesarean deliveries, newborns with birthweight of <2.5 or >4 kg, and newborns diagnosed with congenital anomalies.

### Study population and setting

All women who had normal vaginal delivery between January and April 2018 in the WWRC, previously known as the Women's Hospital in Qatar, had their files and the files of their neonates screened for eligibility. The study was conducted in the NICU of the WWRC during that period.

### Data collection

The Qatar PEARL-Peristat Registry, established in 2016, collects data from hospitals in Qatar offering delivery services, spanning perinatal to postpartum periods. Sponsored by the Hamad Medical Corporation and funded by the Qatar National Research Fund, the registry aims to assess maternal and infant health outcomes and investigate subcohort growth for reproductive health improvement. The Hamad Medical Corporation Institutional Review Board approved the study with a waiver of consent (HMC-IRB 13064/13).

### Data analysis

Patient characteristics and clinical variables of the first 3 groups were analyzed using descriptive analyses. Continuous variables are presented in terms of means and standard deviations, and were compared using analysis of variance, whereas categorical variables are presented in terms of numbers and percentages and were compared using the chi-square test. Multinomial logistic regression was done to assess the associations between different pain management regimens and variables related to neonatal and maternal characteristics and short-term outcomes using the “none” group as the reference group for the analysis.

*P* values were 2-tailed, and *P* values <.05 were considered statistically significant. All statistical analyses were performed using IBM SPSS Statistics for Windows, Version 29.0 (IBM Corp, Armonk, NY).

## Results

The WWRC conducted 22,666 deliveries during the data collection period. Only 7721 of the screened deliveries met the study inclusion criteria. Of those, 114 cases were excluded because of incomplete information. The final sample included 7607 deliveries, classified into the following 4 groups based on pain management regimens assigned at the time of delivery: the no-treatment group (Group 0), those who received EA only (Group 1), IM morphine only (Group 2), or both EA and IM morphine (Group 3).

Table 1 summarizes the baseline maternal characteristics in Groups 0 to 2. The mean maternal age at admission was 28.7, 27.7, and 28.2 years, with gestational age of 39.1, 39.3, and 39.2 weeks at the time of delivery, respectively. Of the 7324 pregnant women, 63.8% were multigravidas, 5.8% were grand multigravidas, and 30.4% were nulligravidas. Nulligravidas formed 28%, 53.5%, and 18.5% of Groups 0, 1, and 2, respectively. A total of 524 women had a vaginal birth after cesarean delivery, with rates of 36%, 43.5%, and 20.4% in Groups 0, 1, and 2, respectively. Most patients had natural pregnancies, whereas 115 mothers had in vitro fertilization pregnancies. The data also include information on the duration of the first and second stages of labor, with the EA group having the longest duration (374.9 and 71 minutes), followed by the IM morphine and “none” groups. Table 2 displays the characteristics of neonates in these 3 study groups. The mean birthweight was 3200 g. Only 56 neonates had a low Apgar score at 1 minute, with the highest number in the IM morphine group (25), followed by the EA group (21) and the “none” group (7). Of the 330 NICU admissions, 189 were for sepsis and 189 were for respiratory distress. The EA group had the highest rate of NICU admissions (167; 6.2%), followed by the IM morphine group (54; 4%) and the “none” group (109; 3.3%).

**Table 1:** Characteristics of women who received epidural and morphine analgesia during labor

Maternal variables	None	Epidural	Morphine	Total	*P* value
	(n=3304)	(n=2682)	(n=1338)	(n=7324)	
Parity					<.001
Null	623 (18.9%)	1189 (44.3%)	411 (30.7%)	2223 (30.4%)	
Multi	2429 (73.5%)	1399 (52.2%)	845 (63.2%)	4673 (63.8%)	
Grand	252 (7.6%)	94 (3.5%)	82 (6.1%)	428 (5.8%)	
VBAC	189 (5.7%)	228 (8.5%)	107 (8%)	524 (7.2%)	<.001
Pregnancy mode					<.001
Natural	3237 (98%)	2606 (97.2%)	1302 (97.3%)	7145 (97.6%)	
IVF	32 (1%)	61 (2.3%)	22 (1.6%)	115 (1.6%)	
Instrumental vaginal delivery	216 (5.8%)	441 (16.4%)	65 (4.9%)	722 (9.9%)	<.001
Intrapartum fever	7 (0.2%)	74 (2.8%)	15 (1.1%)	96 (1.3%)	<.001
Entonox	1141 (34.5%)	327 (12.2%)	453 (33.9%)	1921 (26.2%)	<.001
PROM	153 (4.6%)	314 (11.7%)	102 (7.6%)	569 (7.8%)	<.001
Meconium stained	478 (14.5%)	455 (17%)	208 (15.5%)	1141 (15.6%)	.03
Chorioamnionitis	8 (0.2%)	59 (2.2%)	13 (1%)	80 (1.1%)	<.001
PPH	106 (3.2%)	203 (7.6%)	44 (3.3%)	353 (4.8%)	<.001
Age at admission (y)	28.7±5.3	27.7±5.2	28±5.3	28.2±5.3	<.001
Weight at delivery	75.8±13.8	78.2±14.2	75.8±13.8	76.7±14	<.001
BMI (kg/m^2^) at delivery	30±5.1	30.7±5.3	30±5.2	30.3±5.2	<.001
Duration of labor first-stage labor (min)	296.1±226.6	374.9±175.6	323.8±160.1	330±201	<.001
Duration of labor second-stage labor (min)	19.5±57.3	71.2±111.6	29.8±78.8	40.3±88	<.001
Duration of labor third-stage labor (min)	7.3±27.2	7.3±28.7	17.8±395.7	9.2±171.2	<.001

Continuous variables are presented as means and standard deviations, and were compared using analysis of variance. Categorical variables are presented as numbers and percentages, and were compared using the chi-square test. Descriptive maternal characteristics are shown among the “none,” epidural analgesia, and intramuscular morphine groups. Multi: ≥1 to <4 pregnancies; grand: ≥5 pregnancies. Entonox is a 50:50 mixture of nitrous oxide and oxygen.

*BMI*, body mass index; *IVF*, in vitro fertilization; *PPH*, postpartum hemorrhage; *PROM*, premature rupture of membranes; *VBAC*, vaginal birth after cesarean delivery.

**Table 2:** Characteristics of newborns of women who received epidural and morphine analgesia during labor

Neonatal baseline characteristics	None	Epidural	Morphine	Total	*P* value
	(n=3304)	(n=2682)	(n=1338)	(n=7324)	
NICU admission (total)	109 (3.3%)	167 (6.2%)	54 (4%)	330 (4.5%)	<.001
For sepsis	56 (1.7%)	105 (3.9%)	28 (2.1%)	189 (2.6%)	<.001
For respiratory distress	33 (1%)	47 (1.8%)	12 (0.9%)	92 (1.3%)	.01
Low Apgar (1 min)	7 (0.2 %)	21 (0.8%)	25 (1.9%)	53 (0.7%)	<.001
Low Apgar (5 min)	2 (0.1%)	2 (0.1%)	1 (0.1%)	5 (0.1%)	.97
Intrahospital death	3 (0.1%)	0 (0%)	1 (0.1%)	4 (0.1%)	.31
Sex					.71
Male	1653 (50%)	1370 (51.1%)	679 (50.7%)	3702 (50.5%)	
Female	1651 (50%)	1312 (48.9%)	659 (49.3%)	3622 (49.5%)	
Birth trauma	13 (0.4%)	28 (1%)	15 (1.1%)	56 (0.8%)	.00
Birthweight (g)	3244.7±342	3264±348.5	3253.5±338.9	3253.4±344	.10
Gestational wk	39.2±1.1	39.3±1.1	39.2±1.1	39.2±1.1	.00
Arterial pH	7.3±0.2	7.3±0.1	7.3±0.1	7.3±0.1	.01
Venous pH	7.4±0.1	7.4±0.1	7.4±0.1	7.4±0.1	.02
Arterial base excess (mmol/L)	−2.9±3.8	−3.4±5.2	−2.7±3.9	−3.1±4.5	.19
Venous base excess (mmol/L)	−3.5±5.7	−3.8±5.5	−3.7±3.5	−3.7±5.3	.79

Continuous variables are presented as means and standard deviations, and were compared using analysis of variance. Categorical variables are presented as numbers and percentages, and were compared using the chi-square test. Descriptive neonatal characteristics are shown among the “none,” epidural analgesia, and intramuscular morphine groups. Low Apgar score: <7.

NICU, neonatal intensive care unit.

Table 3 presents the findings from a backward stepwise multinomial logistic regression analysis, which was performed to examine the associations between pain management regimens during active labor and maternal and neonatal variables, using the “neither EA nor IM morphine” group as the reference (Table 1). For the EA group and the IM morphine group, the analysis revealed that NICU admission due to suspected sepsis, respiratory distress, or other reasons was not significantly associated with the use of these regimens during labor. However, NICU admission due to respiratory depression was significantly associated with the combined use of EA and IM morphine during labor (adjusted odds ratio [aOR], 8.63; 95% confidence interval [CI], 1.07–69.46; *P*=.04) in a group of patients who received both EA and IM morphine concurrently, either because they initially refused to have EA and were given IM morphine or because they required both for severe breakthrough pain during labor. In general, the combined use of EA and IM morphine had the highest number of significant associations with multiple neonatal and maternal variables in comparison with the other 2 groups. Among the variables that exhibited a statistically significant relationship with the combined use of EA and IM morphine were gestational age (aOR, 1.86; 95% CI, 1.19–2.90; *P*=.006), infant sex (aOR, 3.73; 95% CI, 1.54–9.01; *P*=.003), age at admission (aOR, 1.16; 95% CI, 1.05–1.27; *P*=.002), arterial pH (aOR, 0.002; 95% CI, 1.4×10^−5^ to 0.43; *P*=.02), and base excess (aOR, 1.12; 95% CI, 1.01–1.24; *P*=.04), in addition to variables that were significantly associated with the use of EA in the EA group, such as pregnancy mode. Conversely, the use of IM morphine was significantly associated with fewer maternal and neonatal complications such as low Apgar score at 1 minute (aOR, 6.29; 95% CI, 1.33–29.83; *P*=.02) and maternal gravidity (aOR, 0.02; 95% CI, 0.66–0.97; *P*=.02).

**Table 3:** Backward stepwise multinomial logistic regression of variables/outcomes associated with different pain management regimens (epidural analgesia, intramuscular morphine, and combined epidural analgesia and intramuscular morphine)

Pain management regimen	Variables	*P* value	aOR	95% confidence interval	
				Lower bound	Upper bound
Epidural analgesia	Gestational age (wk)	.42	1.10	0.88	1.37
	Female infant sex	.33	1.24	0.81	1.91
	Arterial pH	.82	1.30	0.13	12.71
	Arterial base excess (mmol/L)	.98	1.00	0.94	1.06
	Middle Eastern	<.001	2.91	1.78	4.77
	Assisted reproduction	<.001	2,108,026.53	210,473.51	21,113,231.10
	BMI at delivery (kg/m^2^)	.69	1.01	0.97	1.06
	Labor induction with prostaglandin	<.001	4.17	1.83	9.49
	Maternal age at admission (y)	.29	1.03	0.98	1.08
	Gravidity (N)	.76	0.98	0.84	1.14
	Low Apgar score at 1 min (<7)	.29	2.36	0.48	11.76
	NICU admission for respiratory depression	.25	0.31	0.04	2.28
	Duration of first stage of labor (min)	.01	1.00	1.00	1.00
	Duration of second stage of labor (min)	<.001	1.02	1.01	1.02
Intramuscular morphine	Gestational age (wk)	.68	0.95	0.74	1.22
	Female infant sex	.77	1.08	0.66	1.76
	Arterial pH	.71	1.67	0.11	24.65
	Arterial base excess (mmol/L)	.12	1.06	0.99	1.14
	Middle Eastern	.02	1.95	1.13	3.37
	Assisted reproduction	—	10,633,386.62	10,633,386.62	10,633,386.62
	BMI at delivery (kg/m^2^)	.12	1.04	0.99	1.09
	Labor induction with prostaglandin	.17	2.01	0.74	5.43
	Maternal age at admission (y)	.77	1.01	0.95	1.07
	Gravidity (N)	.02	0.80	0.66	0.97
	Low Apgar score at 1 min (<7)	.02	6.29	1.33	29.83
	NICU admission for respiratory depression	.24	0.23	0.02	2.73
	Duration of first stage of labor (min)	.69	1.00	1.00	1.00
	Duration of second stage of labor (min)	.26	1.00	1.00	1.01
Both	Gestational age (wk)	.01	1.86	1.19	2.90
	Female infant sex	.00	3.73	1.54	9.01
	Arterial pH	.02	0.00	0.00	0.43
	Arterial base excess (mmol/L)	.04	1.12	1.01	1.24
	Middle Eastern	.00	4.17	1.61	10.85
	Assisted reproduction	—	1.42	1.42	1.42
	BMI at delivery (kg/m^2^)	.12	0.93	0.85	1.02
	Labor induction with prostaglandin	<.001	7.49	2.33	24.10
	Maternal age at admission (y)	.00	1.16	1.05	1.27
	Gravidity (N)	.07	0.74	0.53	1.03
	Low Apgar score at 1 min (<7)	.98	0.00	0.00	a
	NICU admission for respiratory depression	.04	8.63	1.07	69.46
	Duration of first stage of labor (min)	.07	1.00	1.00	1.01
	Duration of second stage of labor (min)	<.001	1.02	1.01	1.03
The reference category is the “none” group; received neither EA nor IM morphine.					

The backward stepwise multinomial regression results of different pain management regimens are shown. The variables included in the model are NICU admission for suspected sepsis, NICU admission for respiratory distress, NICU admission for other causes, duration of labor (first stage; minutes), duration of labor (second stage; minutes), duration of labor (third stage; minutes), gestational age, intrapartum fever, birthweight, infant sex (M1/F2), arterial pH, arterial base deficit, Qatari nationality, vaginal birth after cesarean delivery, pregnancy mode (natural vs in vitro fertilization), BMI at delivery, labor induction with prostaglandin, instrumental vaginal delivery, maternal age at admission, parity, gravidity, low Apgar score at 1 minute, birth trauma, meconium-stained amniotic fluid, chorioamnionitis, and postpartum hemorrhage.

*aOR*, adjusted odds ratio; *BMI*; body mass index; *EA*, epidural analgesia; *IM*, intramuscular; *NICU*, neonatal intensive care unit.

a Floating point overflow occurred while computing this statistic. Its value is therefore set to system missing.

Moreover, the use of EA and the combined use of EA and IM morphine were associated with a higher rate of labor induction with prostaglandin (aOR, 4.17; 95% CI, 1.83–9.49; *P*<.001; and aOR, 7.49; 95% CI, 2.38–24.10; *P*<.001; respectively). Significantly longer durations of first and second stages of labor were found in the EA group (aOR, 1.002; 95% CI, 1.001–1.004; *P*=.02; and aOR, 1.02; 95% CI, 1.01–1.02; *P*<.001; respectively), whereas only the second stage of labor was significantly longer in the EA and IM morphine group (aOR, 1.02; 95% CI, 1.01–1.03; *P*<.001). The study also found a significantly higher rate of EA use among Middle Eastern patients compared with patients of other ethnicities (aOR, 2.91; 95% CI, 1.76–4.77; *P*<.001). This trend was consistently significant in the IM morphine group (aOR, 1.95; 95% CI, 1.13–3.37; *P*=.02) and the group with combined IM morphine and EA (aOR, 4.17; 95% CI, 1.61–10.85; *P*=.003).

## Comment

### Principal findings

Our study showed that the use of EA alone or IM morphine alone during active labor is not associated with increased risk of major neonatal or maternal complications, including NICU admission, birth trauma, operative vaginal delivery, chorioamnionitis, meconium-stained amniotic fluid, and postpartum hemorrhage. Although the use of EA prolonged labor duration, it was not associated with instrumental vaginal delivery or birth trauma. Use of IM morphine alone was only associated with low Apgar scores at 1 minute. Furthermore, our study included a group that received both EA and IM morphine, which was associated with not only NICU admission due to respiratory depression but also with several other variables, particularly perinatal acidosis.

### Results in the context of what is known

EA is effective in alleviating labor pain, but a review of the literature suggests an increased risk of neonatal adverse effects, such as higher rates of NICU admission.^4^, ^5^, ^6^, ^7^ Most studies have not considered confounding variables that may affect the relationship between EA use and neonatal complications. Our analysis, which controlled for other variables, found that EA use is not associated with higher rates of short-term adverse effects, such as NICU admission due to sepsis or respiratory distress. This is consistent with previous literature findings. A cohort study on 2 populations found that neither EA use nor prolonged second stage of labor influences short-term neonatal mortality.^13^ A meta-analysis of 8 studies, including 4488 patients, reported no clear differences between groups in neonatal outcomes such as NICU admission (relative risk, 1.03; 95% CI, 0.95–1.12), which was deemed as moderate-quality evidence.^3^ Another study showed no significant association between the use of EA and the rates of neonatal complications (such as the risk of NICU admission) in nulliparous and multiparous women after controlling for confounding factors using a multivariable logistic regression model. However, it reported a statistically significant risk of instrumental vaginal delivery in both nulliparous and multiparous women.^14^ It is widely reported in the literature that the use of EA is associated with a significantly prolonged duration of labor; however, whether EA increases the rates of instrumental vaginal delivery is controversial. A meta-analysis that included 7 randomized controlled trials comparing low-concentration epidural infusions with parenteral opioid use during labor concluded that EA use significantly prolonged labor and increased rates of instrumental vaginal delivery compared with opioid administration.^15^ Another systematic review comparing EA with opioids found that EA was associated with a prolonged second stage of labor, but did not affect the progress of labor, and early placement of NA could be permitted.^16^ Our study showed a significantly prolonged duration of labor with the use of EA; however, we found no increased risk of instrumental vaginal delivery with EA use. These results are consistent with the conclusions from a Cochrane meta-analysis of 30 trials, which indicated that a large number of women who received EA underwent instrumental vaginal delivery. However, this evidence was labeled as low-quality evidence, and this finding was not observed after running a post hoc analysis excluding all studies conducted before 2005, suggesting that modern approaches to EA do not influence this outcome.^14^ This conclusion is also supported by the American Society of Anesthesiologists Task Force (2016), which favors the administration of NA in laboring women, regardless of the stage of labor, stating that the effects of EA on labor course and instrumental vaginal delivery are mostly equivocal.^1^ Opioids are well known for their ability to cross the placenta, and have been linked to maternal and neonatal adverse effects in many studies in the literature. However, in our study, morphine use during labor was not associated with neonatal or maternal variables, except for a low Apgar score at 1 minute.^1^

It is crucial to acknowledge that our study did not explore cesarean delivery rates among women undergoing a trial of labor, as all cesarean deliveries were omitted from our analysis. However, existing literature, including a review from the Cochrane Library, has examined the influence of EA on cesarean delivery rates, finding no significant impact.^3^ A study by Salameh et al^4^ in Qatar reported a 22% cesarean delivery rate without a notable difference between women who received EA and those who did not. Furthermore, research from the same institution revealed a 14% rate of emergency cesarean delivery, primarily due to fetal distress, stalled labor, or failed induction,^17^ although the role of analgesia type was not assessed. In addition, studies comparing parenteral opioid administration and EA have also shown no significant difference in cesarean delivery rates.^18^^,^^19^

### Clinical and research implications

This study found a significant increase in NICU admissions due to respiratory depression among women receiving both EA and IM morphine. There is a lack of studies comparing the combined use of EA and IM morphine with no analgesia, limiting the ability to draw comparisons. This gap in the literature highlights the need for further research to assess the potential adverse effects of combining these 2 regimens during labor. The analysis also showed an association between the combined use of EA and IM morphine and arterial pH and arterial base deficit, indicating uncertainty about their safety during active labor. Therefore, caution should be applied with the combined use of both methods of pain control during labor.

### Strengths and limitations

One of the primary strengths of this study is its comprehensive and diverse patient population, including both nulliparous and multigravid mothers with low-risk singleton pregnancies. This broad sample base provides a high level of generalizability to a wider population. The study's methodology and rigorous statistical approach, which categorizes patients into distinct groups based on their analgesic regimen, allow for a detailed understanding of the effects of different analgesic methods on maternal and neonatal outcomes.

In addition, the study benefits from being conducted in a controlled environment, the WWRC, ensuring consistent labor and delivery management practices across the study population. The use of the Qatar PEARL-Peristat Registry for systematic data collection adds to the study's robustness, encompassing a wide range of maternal and infant health outcomes.

One limitation of our study is its retrospective design, which hampers our ability to infer causality and fully account for all confounding factors. Such design depends on preexisting records, which can introduce discrepancies or incomplete data. In addition, retrospective research may not encompass all pertinent details, particularly those related to the timing and specifics of interventions and outcomes.

Furthermore, given that our study was conducted at a single center, it might exhibit selection bias, especially because it primarily included low-risk pregnancies, a decision dictated by the available data. We also did not include information on women who underwent cesarean delivery, those with complex prenatal histories, or those with significant birthweight deviations, which could further limit the generalizability of our findings.

### Conclusion

This study provides valuable insights into the use of EA and IM morphine during labor, particularly in the context of NICU admissions and short-term maternal and neonatal outcomes. Our findings suggest that, although EA and IM morphine resulted in distinct clinical profiles when used alone during labor in low-risk mothers, neither method increased risk of NICU admission. However, the combined use of EA and IM morphine is associated with increased risk of NICU admission due to respiratory depression and other adverse effects, underlining the need for caution in this practice. Future studies should focus on a comprehensive evaluation of the combined use of EA and IM analgesia during labor, especially concerning maternal and neonatal outcomes.

## CRediT authorship contribution statement

**Abdelrahman Elsayed:** Data curation, Methodology, Writing – original draft, Writing – review & editing. **Ismail Abdelhady:** Conceptualization, Data curation, Writing – review & editing. **Fawzia M. Elgharbawy:** Data curation, Writing – review & editing. **Ashraf Gad:** Conceptualization, Data curation, Formal analysis, Writing – review & editing.
